# The Adjuvant Activity of *Epimedium* Polysaccharide-Propolis Flavone Liposome on Enhancing Immune Responses to Inactivated Porcine Circovirus Vaccine in Mice

**DOI:** 10.1155/2015/972083

**Published:** 2015-11-03

**Authors:** Yunpeng Fan, Liwei Guo, Weifeng Hou, Chao Guo, Weimin Zhang, Xia Ma, Lin Ma, Xiaoping Song

**Affiliations:** ^1^College of Veterinary Medicine, Northwest A&F University, Yangling, Shaanxi 712100, China; ^2^College of Animal Science, Yangtze University, Jingzhou 434023, China; ^3^Medicinal Engineering Department of Henan University of Animal Husbandry and Economy, Zhengzhou, Henan 450011, China

## Abstract

*Objectives.* The adjuvant activity of* Epimedium* polysaccharide-propolis flavone liposome (EPL) was investigated in vitro and in vivo.* Methods.* In vitro, the effects of EPL at different concentrations on splenic lymphocytes proliferation and mRNA expression of IFN-*γ* and IL-6 were determined. In vivo, the adjuvant activities of EPL, EP, and mineral oil were compared in BALB/c mice through vaccination with inactivated porcine circovirus type 2 (PCV2) vaccine.* Results.* In vitro, EPL promoted lymphocytes proliferation and increased the mRNA expression of IFN-*γ* and IL-6, and the effect was significantly better than EP at all concentrations. In vivo, EPL significantly promoted the lymphocytes proliferation and the secretion of cytokines and improved the killing activity of NK cells, PCV2-specific antibody titers, and the proportion of T-cell subgroups. The effects of EPL were significantly better than EP and oil adjuvant at most time points.* Conclusion.* EPL could significantly improve both PCV2-specific cellular and humoral immune responses, and its medium dose had the best efficacy. Therefore, EPL would be exploited in an effective immune adjuvant for inactivated PCV2 vaccine.

## 1. Introduction

Porcine circovirus (PCV) is a single-stranded DNA virus, which is one of the smallest animal viruses known to man [1]. There are two genotypes: PCV1 and PCV2. PCV2 possesses pathogenicity, which is the major pathogen for causing postweaning multisystemic wasting syndrome (PMWS) in piglets [2]. Studies show that PCV2 can lead to concurrent infection combined with other viruses such as porcine reproductive and respiratory syndrome virus and porcine parvovirus [3, 4]. Moreover, PCV2 can infringe the lymphatic system of pig and thus induce the deficiency of lymphocytes and antigen presenting cells which result in the immunosuppression of body and easily cause secondary infection [5]. Therefore, how to effectively prevent and control PCV2 infection is the issue to be solved urgently in pig industry. PCV2 has become one of the research hotspots in the field of veterinary medicine at home and abroad.

For PCV2 disease, there is no effective medicine for its treatment in clinic. Vaccination is an effective way for preventing this disease. But the immune response induced by most vaccines is suboptimal without the help of adjuvants. Immune adjuvant plays an essential role in vaccine [6]. However, there are some deficiencies for commonly used adjuvant in clinic [7, 8]. Therefore, it is particularly important to develop new types of immune adjuvants. Nowadays, the focus of drug research has shifted from synthetic drug to natural medicine, and the study of natural products has become a hotspot [9, 10].

*Epimedium*, a famous traditional Chinese herbal medicine, could significantly enhance the immune function of body. The polysaccharide from* Epimedium* has been proved to be the primary active compound responsible for the immunomodulatory effect of this drug [11].* Propolis* has been widely used as the adjuvant of vaccines, because it could induce earlier immune response and longer protection period [12]. Its main compound is flavone. Our previous studies proved that the prescription of* Epimedium* polysaccharide and propolis flavone (EP) could synergistically enhance humoral immunity and cellular immunity of organism and improve the immune effect of inactivated avian influenza and Newcastle disease vaccine [13, 14].

Studies have confirmed that liposome could improve the bioavailability of drugs [15]. Our previous studies have proved that the immunoenhancing activity of the effective constituents from plants was significantly improved after being made into liposome [16, 17]. Recently, we have found that liposome also could further improve the immune enhancement activity of EP and improve the immune response of Newcastle disease vaccine [18, 19]. Therefore, we speculate EP liposome (EPL) could also enhance the immune effect of PCV2 vaccine. In the present study, the adjuvant activity of EPL against inactivated PCV2 vaccine was investigated. The purpose of this study was to investigate whether EPL could enhance the immune response of PCV2 vaccine and select the best dosage for clinical use. This study will provide theoretical support for developing a new type of adjuvant for inactivated PCV2 vaccine.

## 2. Materials and Methods

### 2.1. Reagents

Lymphocyte separation medium was obtained from Tianjin Hanyang Biologicals Technology Co. Ltd. RPMI-1640 and fetal bovine serum (FBS) were obtained from Hyclone (USA). Trypsin (Amresco) was dissolved in 0.25% with calcium and magnesium-free phosphate-buffered saline (CMF-PBS, pH 7.2). Goat anti-mouse monoclonal anti-CD4-FITC and anti-CD8-PE antibodies were supplied by BioLegend Inc. (USA). The 3-(4,5-dimethylthiazol-2-yl)-2,5-diphenyltetrazolium bromide (MTT) and dimethyl sulfoxide (DSMO) were purchased from Sigma-Aldrich Co. LLC. All the other chemicals used were of analytical grade.

### 2.2. Preparation of EPL

*Epimedium* polysaccharide-propolis flavone liposome (EPL) was prepared in our laboratory according to previous method [19]. EPL solution with a concentration of 2.0 mg·mL^−1^ was prepared by dissolving in PBS. The solution was sterilized by a 0.22 *μ*m Millipore filter. The endotoxin amount was up to the standard of the Chinese Veterinary Pharmacopoeia (less than 0.5 EU/mL). Then, it is stored at 4°C for the test.

### 2.3. Vaccine and Virus

Inactivated PCV2 (SH strain) was offered by China Institute of Veterinary Drug Control. The inactivated virus was used as vaccine antigen. EPL adjuvant vaccine at three doses contained 2.0, 1.5, and 1.0 mg of EPL, respectively, in 1 mL of vaccine. EP adjuvant vaccine contained 2.0 mg of EP in 1 mL of vaccine. Oil adjuvant (OA) vaccine was prepared by emulsification with mineral oil according to the literature [20]. All the vaccines contained the same amount of PCV2.

### 2.4. Measurement of Splenic Lymphocytes Proliferation In Vitro

The splenic lymphocytes were prepared according to previous report [21]. 100 *μ*L/well of splenic lymphocytes (5.0 × 10^6^ cells/mL) was plated on a 96-well culture plate. Then, 80 *μ*L of EPL, EP, and blank liposome (BL) in different concentrations was added into each well, respectively. Finally, PHA, LPS, or medium was added for giving a final volume of 200 *μ*L. The final concentration of PHA reached 20 *μ*g·mL^−1^, and LPS reached 10 *μ*g·mL^−1^. Each concentration of sample was repeated four times. The plates were cultured at 37°C in a 5% CO_2_ humidified incubator for 48 h. 20 *μ*L of MTT solution (5 *μ*g·mL^−1^) was added to each well and incubated for an additional 4 h. The supernatant was removed and 100 *μ*L of DMSO was added. The absorbance of cells was evaluated by using microplate reader (Bio-Rad, USA) at a wavelength of 570 nm (*A*_570_ value). The stimulation index (SI) was calculated as the index of splenic lymphocytes proliferation based on the following formula: SI = the absorbance value of drug group/the absorbance value of cell control group [22].

### 2.5. Measurement of IFN-*γ* and IL-6 mRNA Expression In Vitro

The method for preparing splenic lymphocytes was performed according to previous report [21]. After being treated with EPL for 48 h, the RNA of lymphocytes was extracted. The reaction condition was carried out according to the manufacturer's instruction. Real-time quantitative PCR was utilized on the ABI 7300 (PE Applied Biosystems, USA) using SYBR premix Taq Kit (Takara, China). The primers for target gene and GAPDH were listed as follows: IFN-*γ* (183 bp), forward: 5′-CGGCACAGTCATTGAAAGCCTA-3′, reverse: 5′-GTTGCTGATGGCCTGATTGTC-3′; IL-6 (141 bp), forward: 5′-GAGGATACCACTCCCAACAGACC-3′, reverse: 5′-AAGTGCATCATCGTTGTTCATACA-3′; GAPDH (108 bp), forward: 5′-AAATGGTGAAGGTCGGTGTG-3′, reverse: 5′-TGAAGGGGTCGTTGATGG-3′. Amplification was carried out in a final volume of 25 *μ*L. The following experimental run protocol was used: denaturation (95°C for 30 s), annealing (60°C for 30 s), and elongation (72°C for 30 s). The program was performed with 40 PCR cycles. The relative quantification among specimens was calculated according to 2^−ΔΔCt^ method [23].

### 2.6. The Design of Experiment In Vivo

#### 2.6.1. Animals

Six-week-old BALB/c mice weighing 23–25 g were purchased from Laboratory Animal Center of Fourth Military Medical University (Shaanxi, China). They were fed in an environmentally controlled animal facility maintained under controlled conditions with humidity of 50 ± 10%, temperature of 24 ± 1°C, and a 12/12 h light/dark cycle. All the procedures were in strict accordance with the National Institutes of Health Guide for the Care and Use of Experimental Animals approved by the Animal Ethics Committee of Northwest A&F University. All efforts were made to minimize the number of animals used and their suffering.

#### 2.6.2. Adjuvant Activity

Three hundred and sixty BALB/c mice were randomly divided into six groups with 60 mice in each. The mice in three dosage groups (2.0, 1.5, and 1.0 mg·mL^−1^) of EPL were subcutaneously injected with 0.3 mL of EPL vaccine, respectively. In EP and OA groups, the mice were injected with 0.3 mL of EP vaccine and OA vaccine, respectively. In blank control (BC) group, the mice were injected with 0.3 mL of physiological saline. The mice were repeatedly vaccinated after two weeks. On days 7, 14, 21, 28, and 35 after the first vaccination, the blood samples of six mice were collected randomly from each group for measuring peripheral lymphocytes proliferation by MTT method, specific PCV2 antibody titers, and the contents of cytokines (IFN-*γ* and IL-6) in serum by ELISA. At the same time, splenocytes were harvested for determining the killing activity of NK cells. In addition, the blood samples of four mice were collected randomly from each group for assaying CD4^+^ and CD8^+^ T-lymphocytes subpopulations by flow cytometry on days 21, 28, and 35.

#### 2.6.3. Measurement of Cytokines Levels

The contents of IFN-*γ* and IL-6 in serum were assayed by indirect ELISA according to the manufacturer's instructions (Biosamite Biotechnology Co. Ltd., Shanghai, China). The absorbance was measured by using a microplate reader at 450 nm. The contents of IFN-*γ* and IL-6 were calculated according to the standard curve.

#### 2.6.4. Measurement of NK Cell Activity

The killing activity of NK cell in splenocytes was determined by MTT method [24]. Briefly, SP2/0 cells were used as target cells, which were adjusted to 2.0 × 10^4^ cells/mL, and seeded in 96-well culture plates with 100 *μ*L/well. Then, splenocytes were prepared and adjusted to 1.0 × 10^6^ cells/mL and added to a final volume of 200 *μ*L. The plates were incubated for 20 h at 37°C in 5% CO_2_ humidified incubator. 50 *μ*L of MTT solution (5 mg·mL^−1^) was added to each well and the plate was incubated for another 4 h. The absorbance of cells was evaluated at a wavelength of 630 nm (*A*_630_ value). NK cell activity was calculated as the following equation: NK cell activity (%) = (*tA*_630_ − (*sA*_630_ − *bA*_630_))/*tA*_630_ × 100%, where *tA*_630_ is the absorbance of the target cells control, *sA*_630_ is the absorbance of test samples, and *bA*_630_ is the absorbance of blank cells control [24].

#### 2.6.5. Measurement of PCV2-Specific Antibody Titers

PCV2-specific IgG, IgG1, and IgG2a antibodies titers were measured by indirect ELISA according to the manufacturer's instructions. The absorbance was measured in a microplate reader at 630 nm [25]. ELISA assays were performed on the same day for all of the samples.

#### 2.6.6. Measurement of CD4^+^ and CD8^+^ T-Lymphocytes Percentages

Peripheral blood lymphocytes were isolated according to the previous method [26]. The cells were adjusted to 5.0 × 10^6^·mL^−1^. Then, lymphocytes were incubated at 4°C for 30 min in the dark with FITC-conjugated anti-mouse CD4 and PE-conjugated anti-mouse CD8 monoclonal antibodies. After being centrifuged at 3000 ×g for 5 min at 4°C, the cells were washed twice with ice-cold PBS and resuspended in 0.5 mL of PBS. The proportions of CD4^+^ and CD8^+^ T-lymphocytes were assayed by flow cytometry.

### 2.7. Statistical Analysis

The data were expressed as mean ± standard deviation (SD). Duncan's multiple range test was used to determine the differences among groups with the software SPSS 19.0. *P* values of less than 0.05 were considered to be statistically significant.

## 3. Result

### 3.1. Effect of EPL on Splenic Lymphocyte Proliferation In Vitro

**Figure 1:**
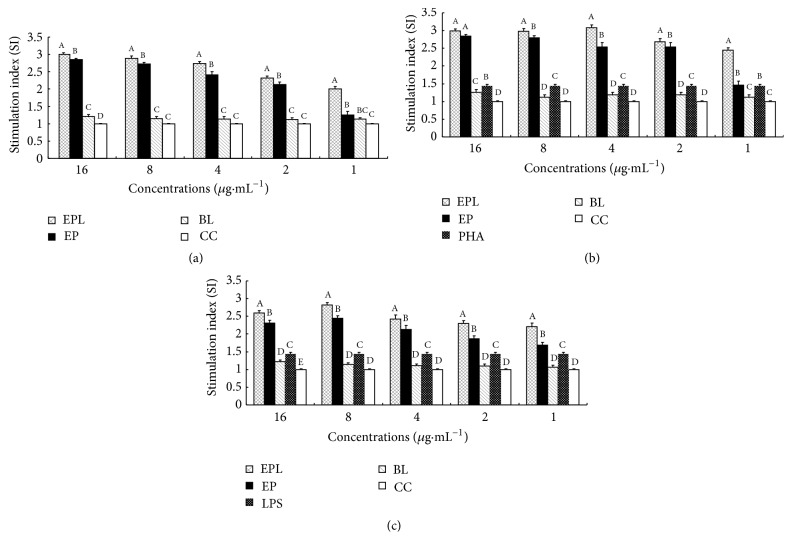
Effect of EPL on splenic lymphocyte proliferation in vitro (SI). (a) The single stimulation of drugs. (b) The synergistic stimulation of drugs with PHA. (c) The synergistic stimulation of drugs with LPS. ^A–D^Bars in the figure without the same superscripts differ significantly (*P* < 0.05).

The stimulation indexes (SI) in each group are illustrated in Figure 1. In single stimulation of EPL and EP on splenic lymphocyte, the SI in EPL group was higher than those in BL and cell control (CC) groups under all concentrations (*P* < 0.05). In addition, the SI in EPL group was significantly higher than those in EP group at all concentrations (*P* < 0.05) (Figure 1(a)). After synergistic stimulation with PHA, the SI in EPL group was the highest and was significantly higher than those in EP group at 8–1 *μ*g·mL^−1^ (*P* < 0.05) (Figure 1(b)). After synergistic stimulation with LPS, the SI in EPL group was the highest and was significantly higher than those in EP group at all concentrations (*P* < 0.05) (Figure 1(c)).

### 3.2. Effect of EPL on the mRNA Expression of IFN-*γ* and IL-6 In Vitro

**Figure 2:**
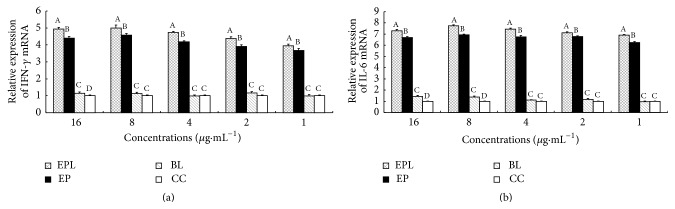
Effect of EPL on the mRNA expression of IFN-*γ* and IL-6. (a) The mRNA expression of IFN-*γ*. (b) The mRNA expression of IL-6. Bars in the figure without the same superscripts differ significantly (*P* < 0.05).

The effects of EPL on the mRNA expression of IFN-*γ* and IL-6 in splenocytes are illustrated in Figure 2. At 16–1 *μ*g·mL^−1^, the expression levels of IFN-*γ* and IL-6 in EPL and EP groups were significantly higher than those in BL and CC groups (*P* < 0.05). In addition, the expression levels of IFN-*γ* and IL-6 in EPL group were significantly higher than that in EP group at all concentrations (*P* < 0.05).

### 3.3. The Dynamic Changes of T-Lymphocytes Proliferation In Vivo

**Figure 3:**
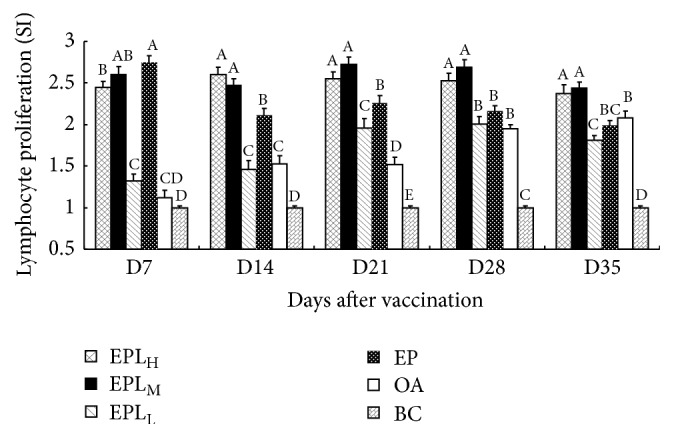
The effects of EPI on T-lymphocytes proliferation in the immunized mice (SI). Bars in the same day without the same superscripts differ significantly (*P* < 0.05). H: high dose; M: medium dose; L: low dose.

The SI in each group is illustrated in Figure 3. At all time points after the first vaccination, the SI in EPL at three doses and EP groups were significantly higher than those in BC group (*P* < 0.05). On days 14–35, the SI in EPL_H_ and EPL_M_ groups were significantly higher than those in EPL_L_ and EP groups (*P* < 0.05). At all time points, the SI in EPL_H_ and EPL_M_ groups were significantly higher than those in OA group (*P* < 0.05).

### 3.4. Effect of EPL on the Secretion of IFN-*γ* and IL-6 in Serum

**Figure 4:**
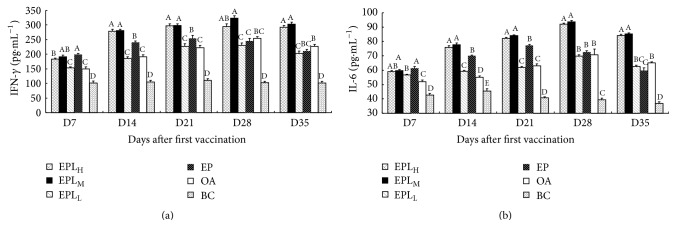
The effects of EPI on the secretion of cytokines in the immunized mice (pg·mL^−1^). (a) The changes of serum IFN-*γ* contents. (b) The changes of serum IL-6 contents. Bars in the same day without the same superscripts differ significantly (*P* < 0.05). H: high dose; M: medium dose; L: low dose.

Effects of EPL on the secretion of IFN-*γ* and IL-6 are shown in Figure 4. On days 14–35, the IFN-*γ* contents in EPL_H_ and EPL_M_ groups were significantly higher than those in EPL_L_ and EP groups (*P* < 0.05). At all time points, the IFN-*γ* contents in EPL_M_ group were significantly higher than those in OA group (*P* < 0.05). The IFN-*γ* contents in the EPL_H_ group were significantly higher than those in OA group on days 7, 14, 21, and 35 (*P* < 0.05) (Figure 4(a)). On days 14–35, the IL-6 contents in EPL_H_ and EPL_M_ group were significantly higher than those in EPL_L_, EP, and BC groups (*P* < 0.05). In addition, at all time points, the IL-6 contents in EPL_H_ and EPL_M_ groups were also significantly higher than those in OA group (*P* < 0.05) (Figure 4(b)).

### 3.5. Effect of EPL on the Activity of NK Cells In Vivo

**Figure 5:**
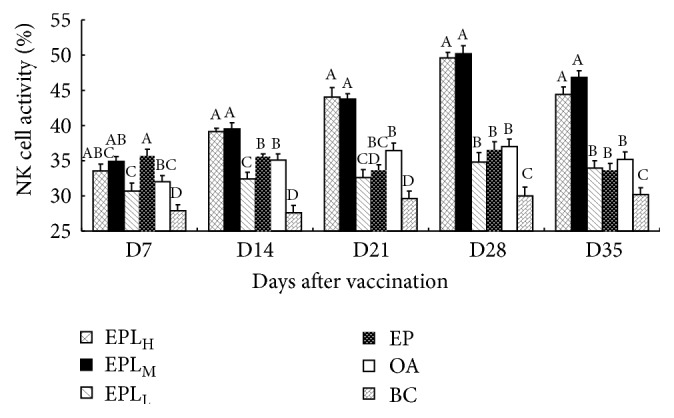
Effect of EPL on the activity of NK cells. Bars in the same day without the same superscripts differ significantly (*P* < 0.05). H: high dose; M: medium dose; L: low dose.

The effects of EPL on NK cell activity were shown in Figure 5. On days 14–35, the activities of NK cells in EPL_H_, EPL_M_, EPL_L_, EP, and OA groups were significantly higher than those in BC group (*P* < 0.05). On days 14–35, the activities of NK cells in EPL_H_ and EPL_M_ groups were significantly higher than those in EPL_L_, EP, and OA groups (*P* < 0.05).

### 3.6. Effect of EPL on the PCV2-Specific Titers

**Figure 6:**
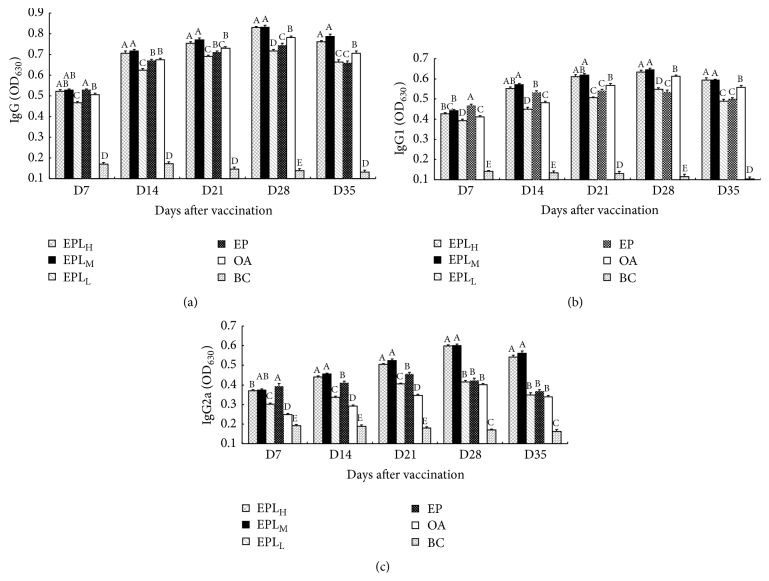
Effect of EPL on PCV2-specific IgG, IgG1, and IgG2a antibodies in the immunized mice. (a) The titer of IgG. (b) The titer of IgG2a. (c) The titer of IgG2a. Bars in the same day without the same superscripts differ significantly (*P* < 0.05). H: high dose; M: medium dose; L: low dose.

The effect of EPL on the PCV2-specific IgG, IgG1, and IgG2a titers was shown in Figure 6. On days 14–35, the IgG titers in EPL_H_ and EPL_M_ groups were significantly higher than those in other groups (*P* < 0.05) (Figure 6(a)). On days 14–35, the IgG1 titers in EPL_M_ group were significantly higher than those in EPL_L_ and EP groups; EPL_H_ group was higher than EP group, and the differences were significant on days 21, 28, and 35 (*P* < 0.05). In addition, at all time points, the IgG1 titers in EPL_M_ group were significantly higher than those in OA group (*P* < 0.05). EPL_H_ was higher than OA, and the differences were significant on days 14, 28, and 35 (*P* < 0.05) (Figure 6(b)). On days 14–35, the IgG2a titers in EPL_H_ and EPL_M_ groups were significantly higher than those in EPL_L_, EP, and OA groups (*P* < 0.05) (Figure 6(c)).

### 3.7. The Effects of EPI on the Percentages of CD4^+^ and CD8^+^ T-Cells

**Figure 7:**
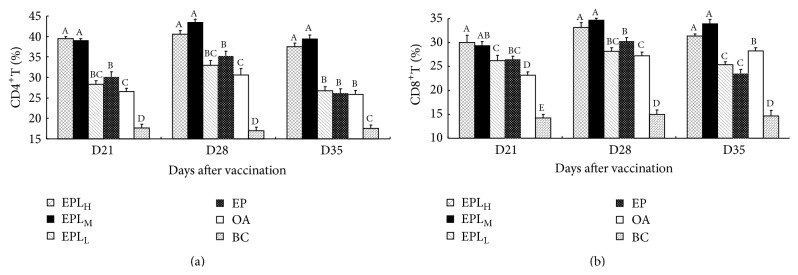
The effects of EPI on the percentage of CD4^+^ and CD8^+^ T-cells in the immunized mice. (a) The changes of CD4^+^ T-cells. (b) The changes of CD8^+^ T-cells. Bars in the same day without the same superscripts differ significantly (*P* < 0.05). H: high dose; M: medium dose; L: low dose.

The changes of CD4^+^ and CD8^+^ T-cells proportions are shown in Figure 7. On days 21–35, the proportions of CD4^+^ T-cells in EPL_H_ group were 39.45%, 40.60%, and 37.53%, in EPL_M_ group were 39.00%, 43.46%, and 39.37%, in EP group were 30.14%, 35.27%, and 26.14%, and in OA group were 26.55%, 30.62%, and 25.88%, respectively. On days 21–35, the proportions of CD4^+^ T-cells in EPL_H_ and EPL_M_ groups were significantly higher than those in EP group (*P* < 0.05). In addition, at all time points, the proportions of CD4^+^ T-cells in EPL_H_ and EPL_M_ groups were significantly higher than those in OA group (*P* < 0.05) (Figure 7(a)). On days 21–35, the proportions of CD8^+^ T-cells in EPL_H_ group were 30.04%, 33.17%, and 31.39%, in EPL_M_ group were 29.31%, 34.68%, and 33.90%, in EP group were 26.51%, 30.26%, and 23.50%, and in OA group were 23.15%, 27.26%, and 28.22%, respectively. On days 21–35, the proportion of CD8^+^ T-cells in EPL_H_ group was significantly higher than those in EP, EPL_L_, and BC groups (*P* < 0.05). At three time points, the proportion of CD8^+^ T-cells in EPL_M_ group was higher than those in EP group, and the differences were significant on days 28 and 35 (*P* < 0.05). In addition, the proportions of CD8^+^ T-cells in EPL_H_ and EPL_M_ groups were significantly higher than those in OA group at all time points (*P* < 0.05) (Figure 7(b)).

## 4. Discussion

The immune system of body mainly consists of cell immunity and humoral immunity [27]. The capacity to induce effective cellular immunity could be determined by T- and B-lymphocytes proliferation [28, 29]. In order to investigate the effect of EPL on enhancing cellular immunity, the splenic lymphocytes proliferation was firstly measured in vitro. The results showed that EPL could significantly promote the splenic lymphocytes proliferation when stimulated with PHA/LPS or single stimulation at 16–1 *μ*g·mL^−1^ compared with EP. In addition, the experimental results in vivo also showed that EPL at high and medium doses could significantly promote peripheral lymphocytes proliferation at most time points. It is suggested that EPL could significantly enhance the activation of T- and B-cells in immunized mice. T-lymphocyte can be divided into CD4^+^ and CD8^+^ subsets [30]. The number of CD4^+^ T-cells and CD8^+^ T-cells is one of the bases in assessing the immune status of organism [31]. The function of cellular immunity and humoral immunity will be improved along with the increasing counts of CD4^+^ and CD8^+^ T-cells [32]. In order to further investigate the effect of EPL on the cellular immunity, the expressions of CD4^+^ and CD8^+^ T-lymphocytes subpopulations were determined. The results showed that the proportions of CD4^+^ and CD8^+^ T-cells in EPL at medium dose group were significantly higher than those in EP group at all time points, which suggested that EPL could promote lymphocytes proliferation and increase the activity of T-cells, thus enhancing the cellular immunity. Related studies have demonstrated that CD4^+^ T-cells play an important role in resisting the pathogens that evade MHC-I processing pathway in acute or chronic infectious diseases [33]. In addition, when CD8^+^ T-cells and irradiated peripheral blood mononuclear cells were cocultivated, some antiviral bioactive substances were found in cell culture supernatant [34]. Therefore, the increasing counts of CD4^+^ and CD8^+^ T-cells will enhance the body's immune and antiviral level. Our previous studies also proved that some effective constituents from plant could improve the proportions of T-lymphocytes subset when encapsulated with liposome [26, 35].

Cytokine is one kind of nonspecific immunoreactive product [36]. IFN-*γ* could promote the proliferation and differentiation of T-cells, activate macrophages, and enhance the cytotoxic effect of NK cells. IL-6 could induce the proliferation and differentiation of B-cells, promote the generation of antibody, and thus mediate humoral immunity [37, 38]. Based on the immunomodulatory effects of IFN-*γ* and IL-6, the effect of EPL on the secretion of IFN-*γ* and IL-6 was measured in this study. The array data showed that EPL could significantly promote the mRNA expression levels of IFN-*γ* and IL-6 in vitro. In addition, the contents of IFN-*γ* and IL-6 in EPL high and medium dosage groups were significantly higher than those in EP group on days 14–35. This suggested that EPL was capable of inducing a large pool of cytokines, and the effect was significantly better than EP. The supplement of EPL in the vaccine is a benefit to improve the immunologic response of body against PCV2.

NK cells are one kind of independent lymphocyte population; they could kill target cells without prior sensitization by antigen. NK cells are the important components of innate immune system and are the first line of defense against the infection of pathogenic microorganism and tumor [39]. Besides killing target cells, NK cells could also release a variety of cytokines (e.g., TNF and IFN-*γ*), which play important roles in the immune regulation and the differentiation of immune cells [40]. In this investigation, the results showed that EPL at high and medium doses and EP could significantly increase the killing activity of NK cells in the immunized mice, and the effect of EPL was significantly superior to EP, suggesting that the usage of EPL in PCV2 vaccine can help to improve the nonspecific immunity to kill virus. Sun et al. also proved that* Albizia julibrissin* saponins from plant could enhance the killing activity of NK cells in the OVA-immunized mice, which is similar to our results [41].

IgG is one of the important immunoglobulins produced in secondary humoral immune response, which possesses various activities, such as antibacterial, antivirus, and antiexotoxin activities. Therefore, the content of IgG not only represents the antibody activity, but also is the characterization reflected by the humoral immune function of body [42]. There are different IgG subclasses including IgG1, IgG2a, IgG2b, and IgG3. The production of IgG subclasses is regulated by T-lymphocytes and their cytokines. IL-4 and IL-6 produced by Th2 lymphocytes improve the production of IgG1, while Th1 cytokines such as IL-2 and IFN-*γ* improve the secretion of IgG2a [43]. According to the immune response of body against the infectious diseases, the Th1 response mainly targets intracellular pathogens, while the Th2 response primarily targets extracellular pathogens [43, 44]. Data in Figure 6 indicated that the EPL could significantly improve the titers of IgG, IgG1, and IgG2a at most time points compared with EP. Enhanced production of IgG and subclasses may be explained by increased secretion of both IFN-*γ* and IL-6 as shown in Figure 4. All these suggested that EPL as an adjuvant could upregulate both Th1 and Th2 immune responses.

PMWS is the serious viral disease and causes a major threat to the pig-breeding industry. Therefore, the development of new efficient adjuvants for PCV2 vaccine is of special urgency. In this study, our results confirmed that EPL could improve the immune response of the inactivated PCV2 vaccine, and the adjuvant effect was significantly better than OA, especially in cellular immunity. In addition, EPL had no side effect, which was safe. Therefore, EPL should be an ideal adjuvant candidate for developing a new type of PCV2 vaccine.

## 5. Conclusions

The present study demonstrated that EPL possessed better adjuvant activity in inducing the specific cellular and humoral immune responses in mice. These findings indicated that EPL may be a promising adjuvant to the inactivated PCV2 vaccine by providing higher and long lasting protective effect. To understand the potential of EPL in clinical applications as adjuvant, further studies on the mechanism of action are in progress.

## List of Abbreviations

PCV: Porcine circovirus
PMWS: Postweaning multisystemic wasting syndrome
EPL: *Epimedium* polysaccharide-propolis flavone liposome
EP: *Epimedium* polysaccharide-propolis flavone
MTT: 3-(4,5-Dimethylthiazol-2-yl)-2,5-diphenyltetrazolium bromide
PBS: Phosphate-buffered saline
PHA: Phytohemagglutinin
LPS: Lipopolysaccharide
DMSO: Dimethyl sulfoxide
BL: Blank liposome
CC: Cell control
OA: Oil adjuvant
BC: Blank control.
